# Revisiting Heinz-Lippman disease as a complication of chronic venous insufficiency

**DOI:** 10.1016/j.jvscit.2023.101408

**Published:** 2023-12-29

**Authors:** Alisha Oropallo, Amanda Beneat, Amit Rao, Eric Goodman

**Affiliations:** a Donald and Barbara Zucker School of Medicine at Hofstra/Northwell, Hempstead, NY; b Department of Vascular Surgery, Northwell Health, Manhasset, NY; c Northwell Health Comprehensive Wound Care Center, North Shore University Hospital, Lake Success, NY; d Department of Radiology, Northwell Health, Manhasset, NY

**Keywords:** Calcification, Computed tomography, Fluorescent imaging, Heterotopic ossification, Venous insufficiency

## Abstract

Chronic venous insufficiency with and without ulceration is a common complication of varicose veins. Heinz-Lippmann disease is a potentially underreported complication of chronic venous insufficiency that involves heterotopic ossification of the peri-wound that might contribute to the chronicity of venous leg ulcers. Most commonly, heterotopic ossification is associated with traumatic injury. We report the cases of three patients with chronic venous insufficiency and no history of trauma who were found to have subcutaneous calcifications consistent with Heinz-Lippmann disease. Osteomyelitis was confirmed in two patients. Physical examination and diagnostic imaging can be helpful in determining the diagnosis. Interventions can include biopsy to rule out osteomyelitis, with subsequent treatment, if needed.

Initially reported in 1960, Heinz, Lippmann, and Goldin found subcutaneous bone formation in 10% of patients with chronic venous insufficiency.^1^ Heterotopic ossification (HO) or Heinz-Lippmann disease is an underrecognized complication of chronic venous insufficiency that might be consistent with venous leg ulcers. Abnormal bone formation and osteoblastic activity is found within any site of connective tissues, including muscle and soft tissues, without connection to the periosteum. The most common locations of HO include the elbow, shoulder, pelvis, and thigh.^2^ Neurogenic forms of HO, often seen after spinal cord and brain injuries, can also be found around major joints.^3^ HO is commonly reported in orthopedics after tissue injury or surgery and has a high prevalence (65%) in combat-related amputations.^4^^,^^5^ After injury, an influx of inflammatory cells and downstream signaling of mesenchymal cells inappropriately activate an osteogenic process, which leads to calcification and bone formation in the soft tissues.^6^

Nongenetic forms of HO are the more prevalent; however, rare genetic forms of HO also exist.^6^ HO can be associated with a rare childhood, slowly progressive disorder, fibrodysplasia ossificans progressiva, consisting of multiple congenital skeletal malformations caused by *ACVR1* mutations.^7^ Biopsy can worsen the clinical condition by leading to additional spread of heterotopic lesions.^6^ Progressive osseous heteroplasia, another genetic form of HO, is associated with the *GNAS1* gene.^8^ These lesions favor the skin and subcutaneous tissue.^6^

HO on radiographs is seen as small “eggshell calcifications” that are often represented by palpable hard fixated nodules underneath the skin in patients with chronic venous insufficiency.^6^ Genetic forms of HO will include a more extensive and advanced ossification with a “cocoon-like web” of HO involved in the connective tissues from the dermis to the skeletal muscles.^9^ Computed tomography (CT) can demonstrate deeper underlying ossification or a low-density mass not appreciated on physical examination and is helpful to assist in evaluation of the extent of disease. Magnetic resonance imaging findings can vary depending on the stage of maturation and can help with differentiating HO from other malignancies such as sarcoma, which can have calcifications. Positron emission tomography with CT using radiolabeled fluoride (F18) can detect areas of bone formation in nongenetic HO and the genetic HO active state.^10^ Near-infrared imaging and high-frequency spectral ultrasound are promising for early detection.^11^^,^^12^

The management and treatment of HO are limited. Site-specific classifications have been developed to note the severity of HO inclusive of the clinical and radiographic severity.^13^ Prophylactic strategies commonly include low-dose radiation and nonsteroidal anti-inflammatory drugs, with corticosteroids usually reserved for genetic cases of HO.^5^^,^^6^ Low-dose radiation carries concern for joint stiffness, malignant transformation, and fracture complications, such as nonunion.^14^ Treatment strategies include physical therapy and surgical resection. To date and to the best of our knowledge, no cases have been reported of osteomyelitis in the heterotopic bone. We describe the cases of three patients with Heinz-Lippmann disease, diagnosed using various imaging modalities. Two of the three patients were noted to have acute osteomyelitis of the heterotopic bone on pathologic analysis, and one patient presented with a misdiagnosis of her disease. All three patients provided written informed consent for the report of their case details and imaging studies.

## Case report

### Patient 1

**Fig 1:**
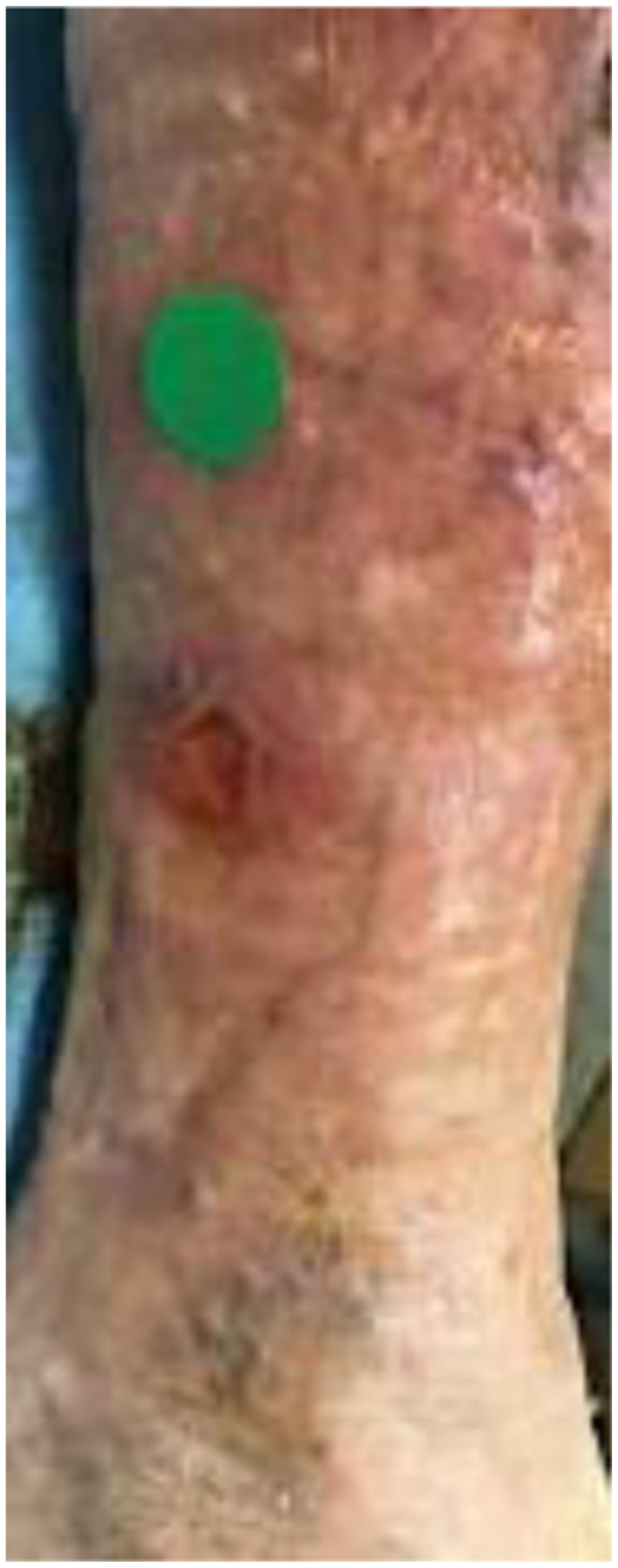
Plain photograph of chronic venous stasis ulcer in a 68-year-old man.

**Fig 2:**
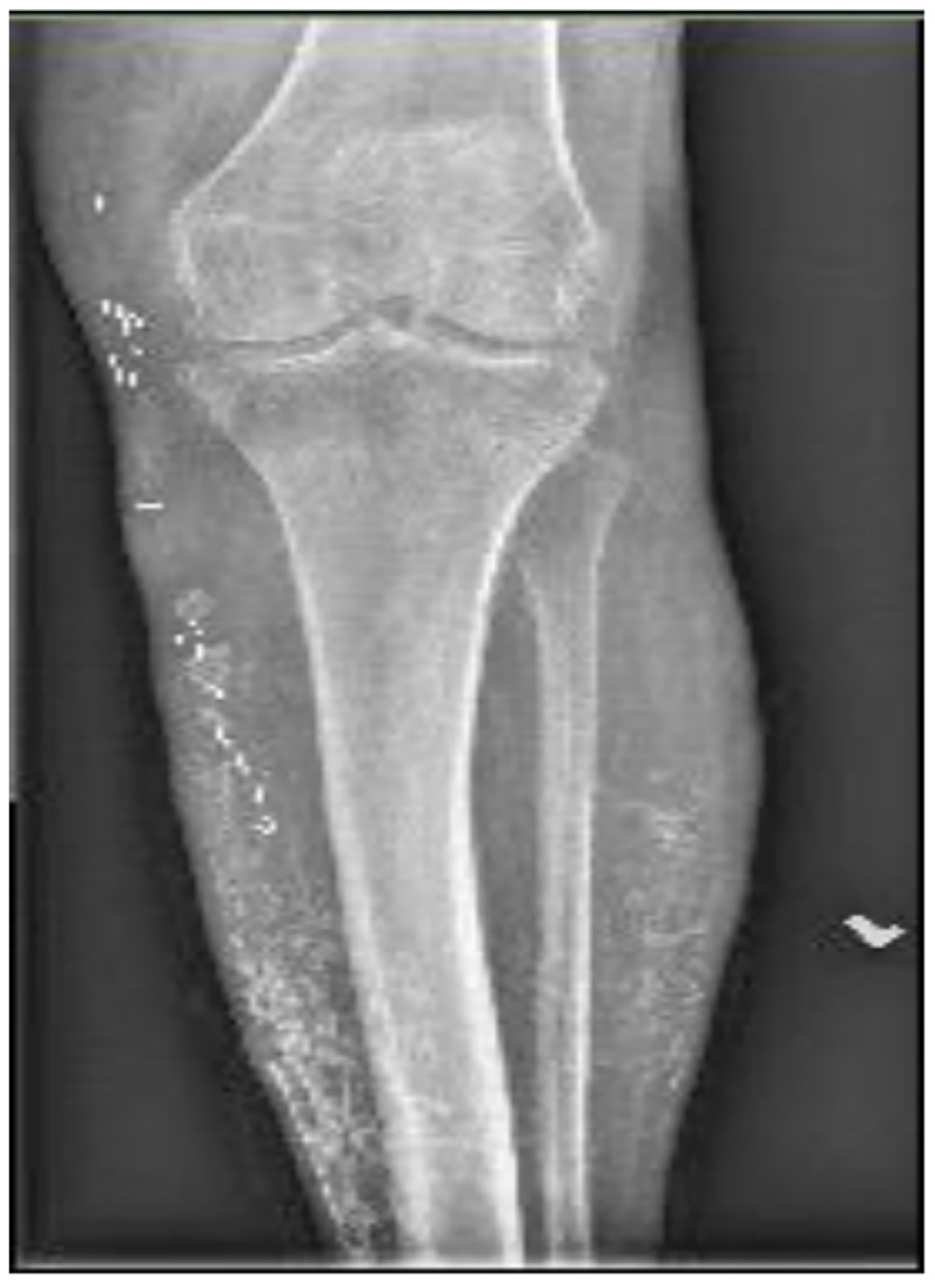
Plain radiograph showing long segments of calcifications, consistent with Heinz-Lippmann disease.

**Fig 3:**
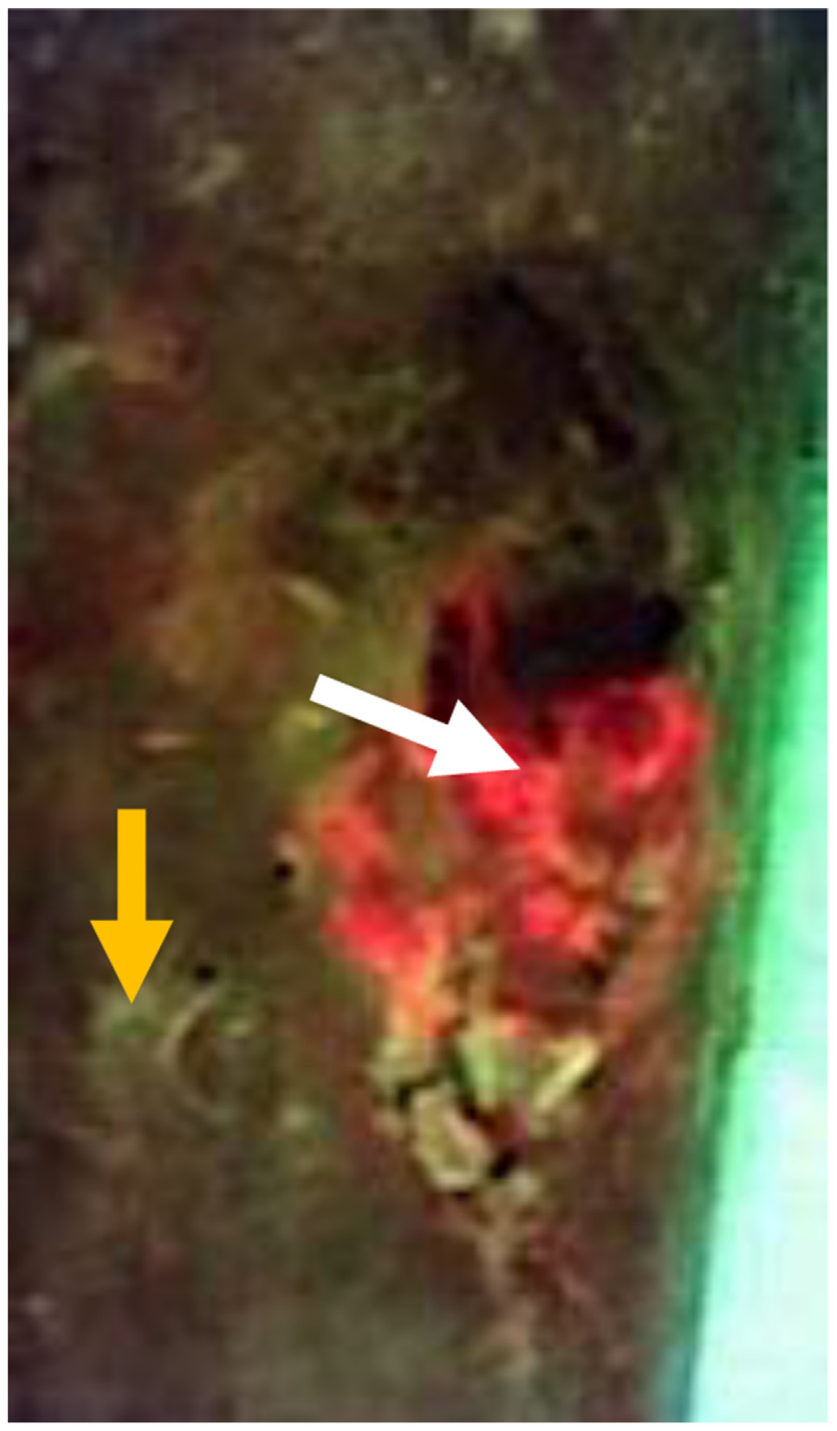
Fluorescent image taken with MolecuLight camera showing areas of red fluorescence (*white arrow*) consistent with *S. aureus* and small, punctate areas of fluorescence (*yellow arrow*) consistent with palpable calcifications.

A 68-year-old man had a history of renal transplant secondary to chronic renal insufficiency, pulmonary hypertension, obstructive sleep apnea, essential hypertension, hyperlipidemia, controlled diabetes mellitus type 2 with neuropathy, and coronary artery disease after coronary stenting. He had been taking daily prednisone 2.5 mg for >1 year’s duration. He presented with edema of the lower extremities, varicosities, and a nonhealing wound (Fig 1). His numeric pain rating scale score was 5 of 10. Physical examination revealed intact pulses with palpable nodules noted underneath the skin to the peri-wound, which were confirmed by radiography (Fig 2). Fluorescence imaging revealed calcifications and red fluorescence, which suggested local infection with *Staphylococcus aureus* (Fig 3). Local wound care involved excisional and mechanical debridement with biopsy of the calcification. Pathologic examination of the biopsy specimen revealed calcification without evidence of osteomyelitis. A standing venous reflux study demonstrated significant reflux of >500 ms of the great saphenous vein. The patient was compliant to compression therapy and underwent radiofrequency ablation. Improvement in the wound size was noted but without complete wound closure. The patient declined further wide excision and skin grafting.

### Patient 2

**Fig 4:**
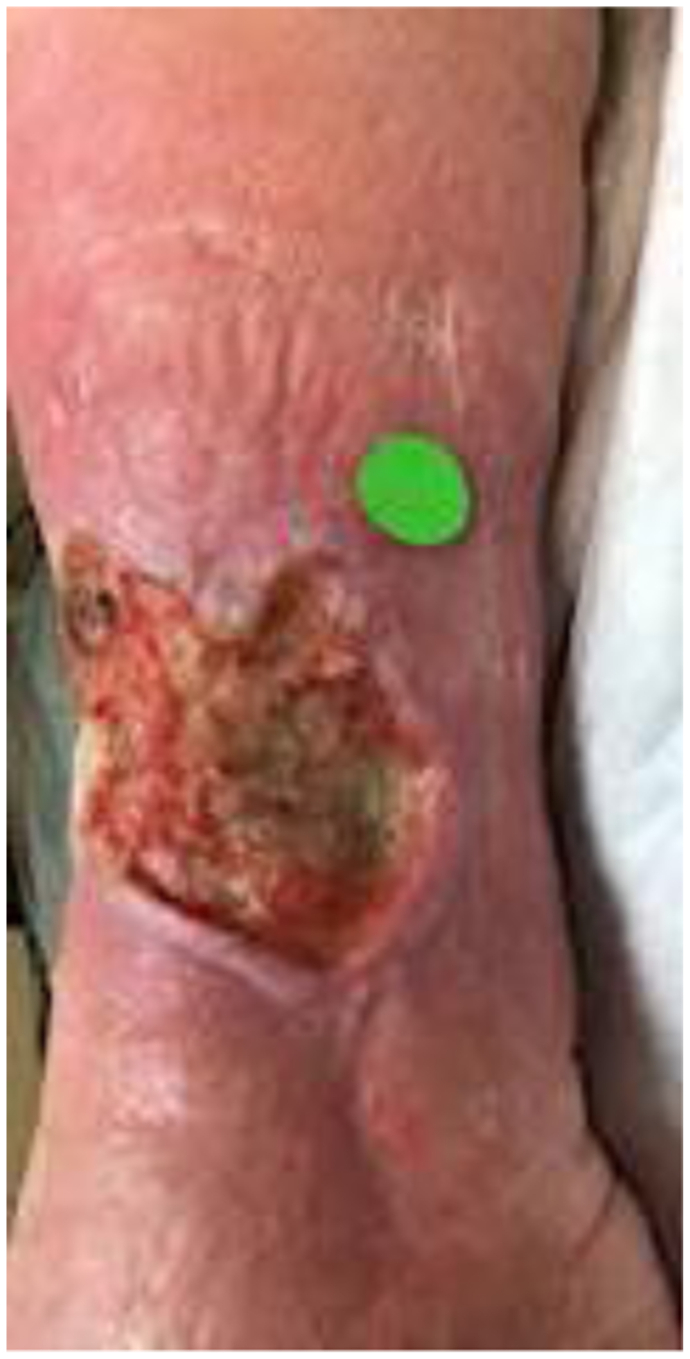
Plain photograph of chronic venous stasis ulcer in a 64-year-old woman.

**Fig 5:**
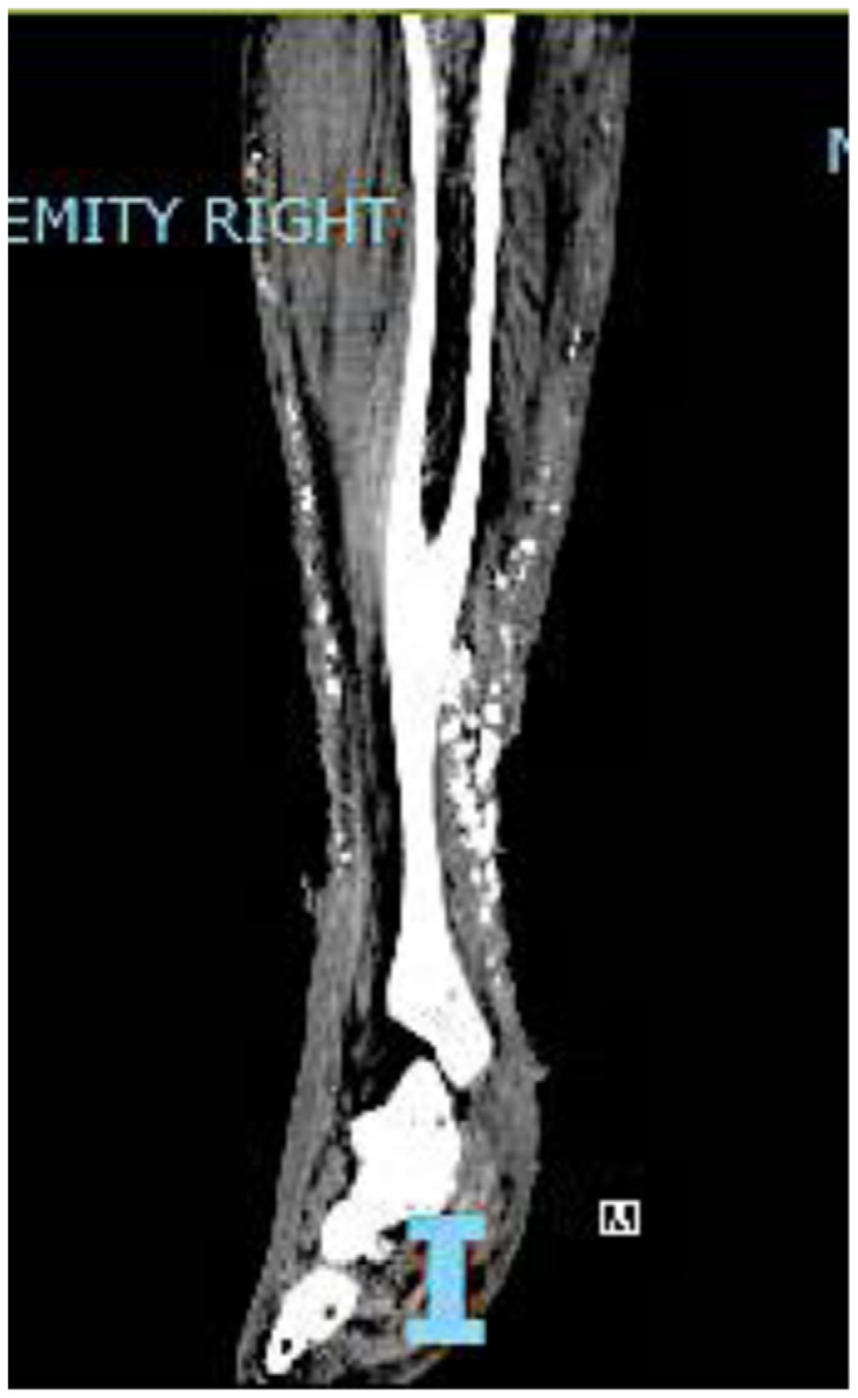
Cross-sectional computed tomography (CT) image showing extensive heterotopic ossification (HO) of the right lower extremity.

**Fig 6:**
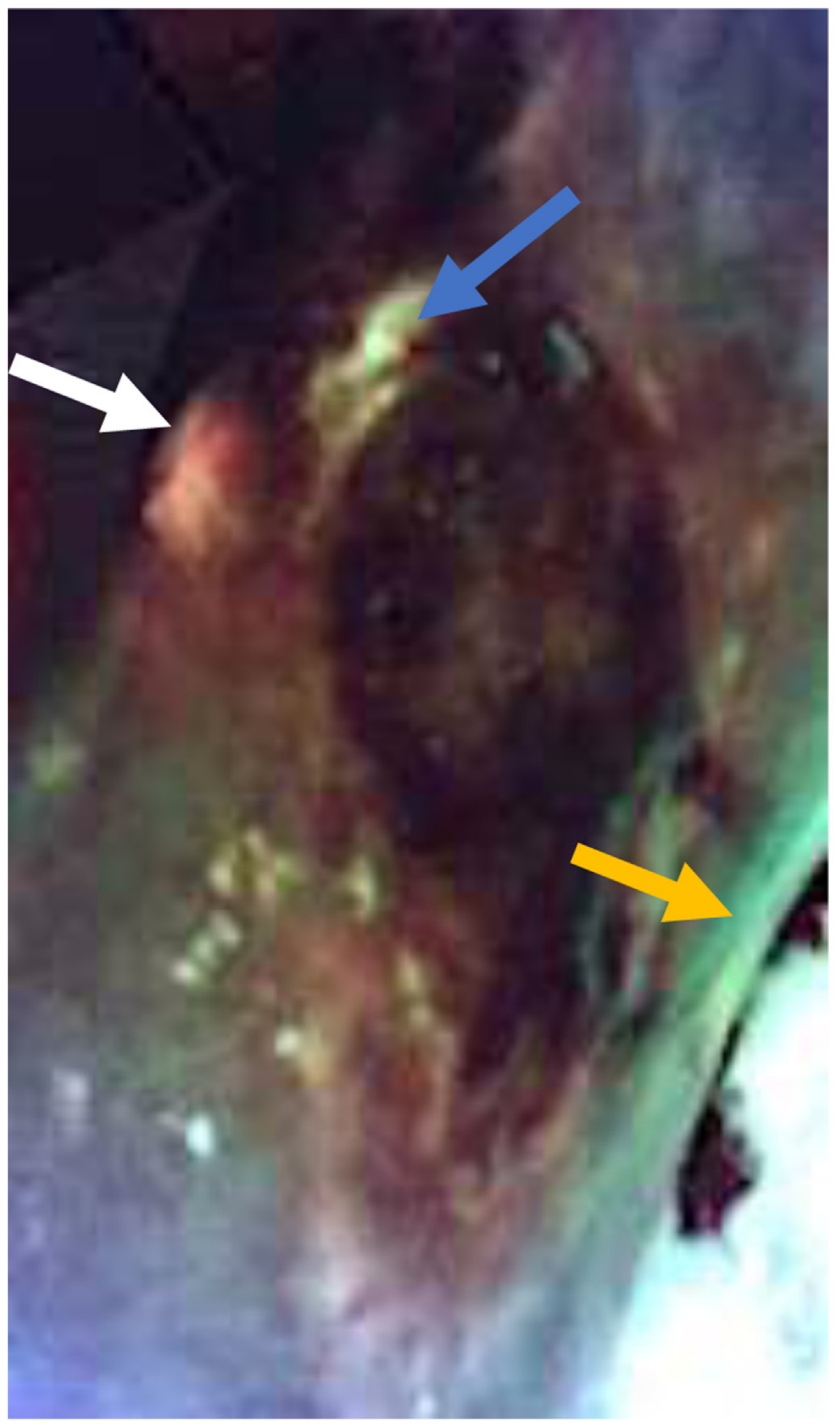
MolecuLight image showing red fluorescence consistent with *S. aureus* (*white arrow*), cyan fluorescence consistent with *P. aeruginosa* (*blue arrow*), and punctate areas of fluorescence (*yellow arrow*) consistent with palpable calcifications.

**Fig 7:**
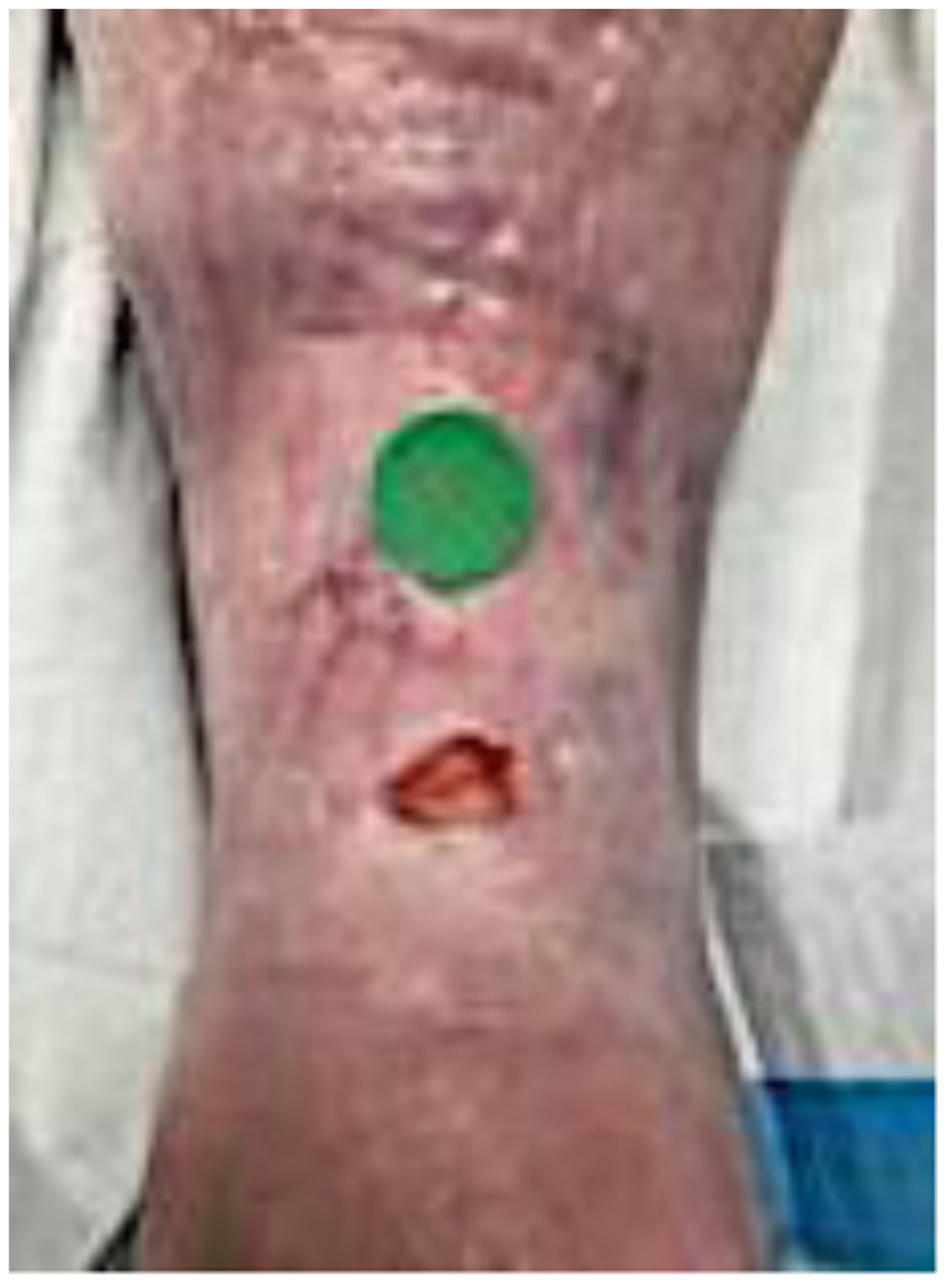
Significant decrease in wound size in patient 2 after treatment with antibiotics, steroids, and radiofrequency ablation.

A 64-year-old woman with a history of chronic venous insufficiency, type 2 diabetes mellitus with neuropathy, hypertension, anemia, and obesity presented with a nonhealing venous leg ulcer of >1 year’s duration (Fig 4). She presented with a previous clinical diagnosis of pyoderma gangrenosum. Her symptoms included pain out of proportion to the physical findings with a numeric pain rating scale score of 10 of 10. Physical examination revealed palpable calcifications within the wound that could be seen on CT (Fig 5). Biopsy of the calcification was noted on pathologic examination to be consistent with HO with acute osteomyelitis. Fluorescent imaging demonstrated multiple calcifications of the peri-wound with underlying *S. aureus* and *Pseudomonas aeruginosa* (Fig 6). Culture confirmed *P. aeruginosa*, *Enterococcus faecalis*, and *S. aureus*. The patient was treated with trimethoprim/sulfamethoxazole (Bactrim DS; Hoffman-La Roche) and cephalexin 500 mg daily. Also, 60 mg of prednisone was initiated, with a 3-week taper to 5 mg daily. Her symptoms of pain resolved. A venous reflux study demonstrated significant reflux of >500 ms to the great saphenous vein, which underwent subsequent radiofrequency ablation. Improvement of the wound size without complete closure was noted (Fig 7). The patient declined further surgical intervention with skin grafting.

### Patient 3

**Fig 8:**
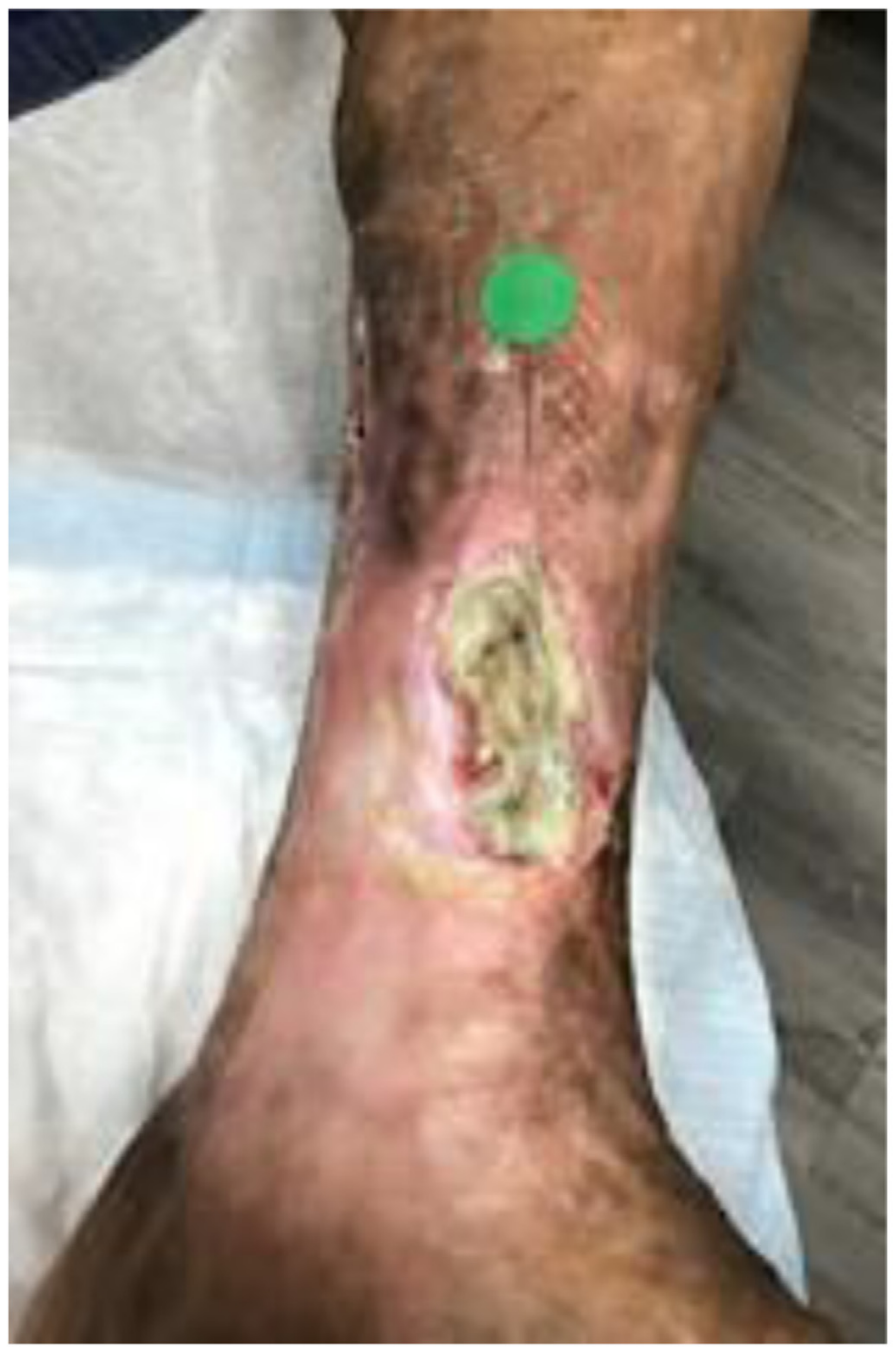
Plain photograph of chronic venous stasis ulcer in a 63-year-old woman.

**Fig 9:**
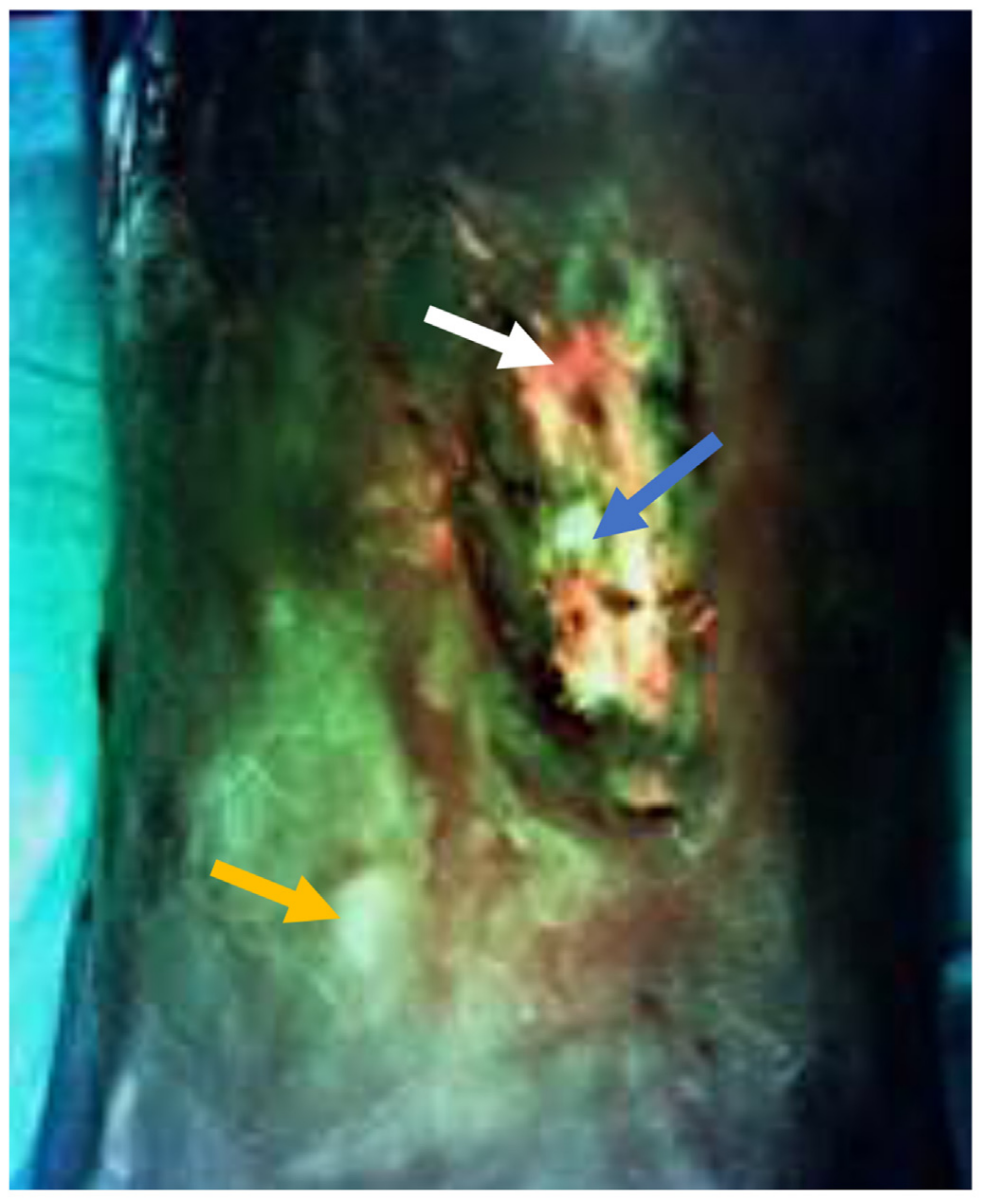
MolecuLight image showing red fluorescence consistent with *S. aureus* (*white arrow*), cyan fluorescence consistent with *P. aeruginosa* (*blue arrow*), and punctate areas of fluorescence (*yellow arrow*) consistent with palpable calcifications.

**Fig 10:**
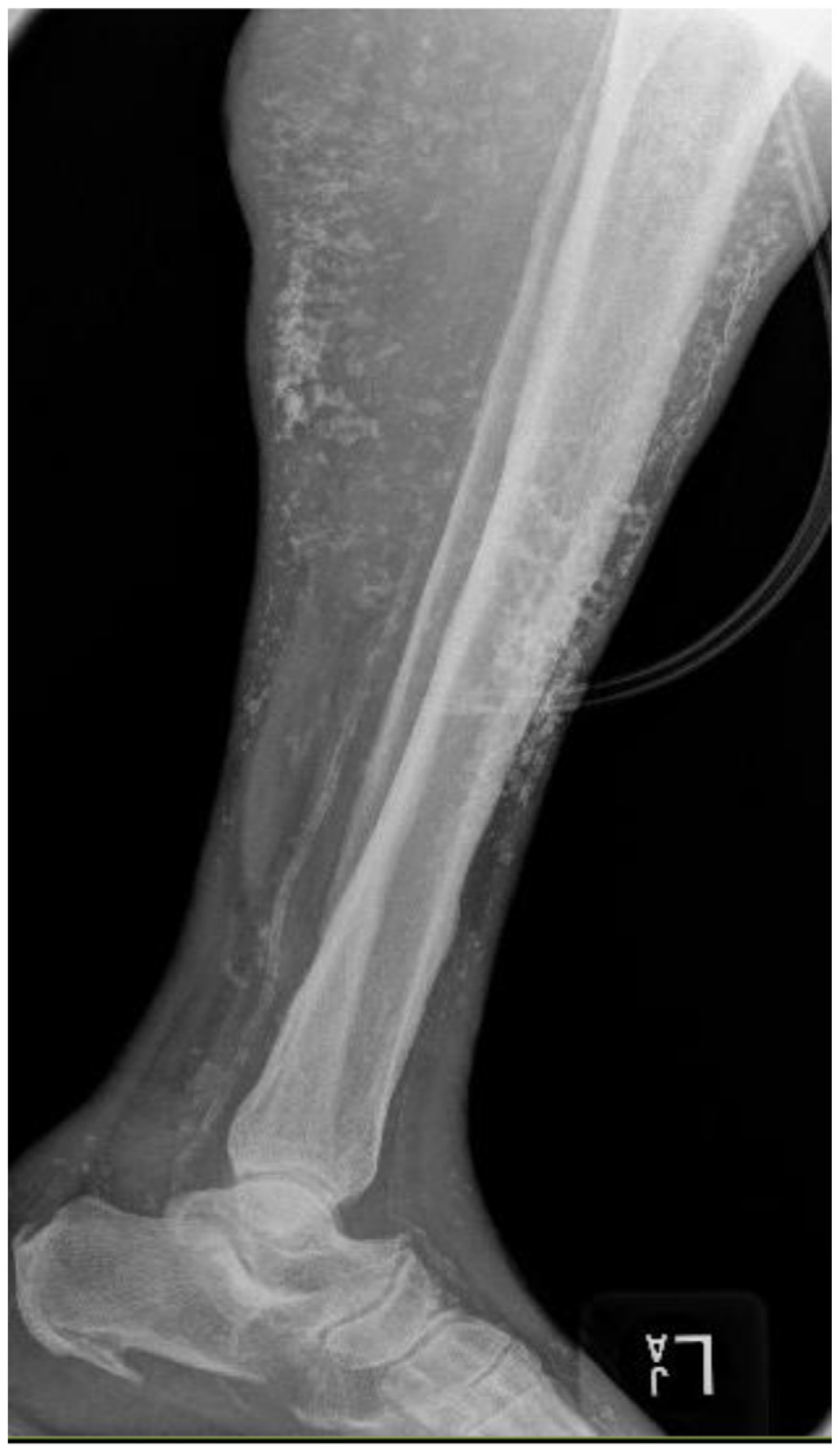
Plain radiograph showing long segments of calcifications in the affected extremity.

A 63-year-old woman presented with a history of a venous leg ulcer of >1 year’s duration with a concomitant diabetic foot ulcer (Fig 8). She had a history of hypertension, hyperlipidemia, osteoarthritis, anemia, neuropathy, and obesity. Her surgical history included sclerotherapy of the left lower extremity. Her pain score was 10 of 10 on a numeric rating scale for pain assessment. Her physical examination demonstrated intact pulses with palpable nodules to the peri-wound of the leg ulcer. Prior venography revealed perforator vessels feeding into the ulcer, and reflux studies had demonstrated significant venous reflux of the great saphenous vein, and she had undergone sclerotherapy. Fluorescent imaging demonstrated calcifications to the peri-wound and the presence of *S. aureus* and *P. aeruginosa* (Fig 9). Culture noted findings consistent with *S. aureus* and *P. aeruginosa*, and the patient was appropriately treated with antibiotics. Plain radiographs of the right lower extremity demonstrated soft tissue calcifications with a calcified venous thrombus (Fig 10). Atherosclerotic calcifications were also noted. A follow-up CT scan revealed extensive bilateral calcifications in the dermis and subcutaneous tissue of both lower extremities, and atherosclerosis of the femoral and popliteal arteries without significant arterial stenosis. The patient's pain was managed, and local wound hygiene with excisional and mechanical debridement was performed. Pathologic examination of the biopsy of the calcification revealed findings consistent with HO and inflammation consistent with acute osteomyelitis. The patient was noncompliant to compression therapy, and her wound healing has been poor.

## Discussion

Classically, HO presents in a young adult, usually male, with a history of trauma or orthopedic surgery.^6^ However, our patients were predominantly postmenopausal women with chronic venous insufficiency and no reported history of trauma. Concomitant autoimmune disorders such as rheumatoid arthritis, CREST (calcinosis, Raynaud's phenomenon, esophageal dysfunction, sclerodactyly, telangiectasia) syndrome, dermatomyositis, and lupus have been reported in association with nontraumatic HO.^6^ Comorbidities such as hypertension, hyperlipidemia, diabetes, obesity, and anemia are also reported and were also prevalent in our patients.

A total of eight case reports were reviewed after a search of the PubMed and MEDLINE databases. Of these, one report noted a history of trauma or burns for the affected patients, and a second reported a history of tibial fracture in the affected leg.^1^ The remaining patients had no reported trauma. Several reports noted pathologic findings consistent with heterotopic bone formation.^15^^,^^16^ All eight reports noted various imaging modalities used in conjunction with the clinical examination, including radiography and CT.

Venous insufficiency, which is caused by poor valvular compliance of the deep and superficial venous systems, leads to reflux of blood and swelling of the lower extremities.^17^ This swelling, if left untreated, can lead to chronic inflammation and mechanical stress, which can result in venous stasis ulcers. The lack of a traumatic or surgical event as the inciting factor for ossification in Heinz-Lippman disease is thought to be due to the presence of this chronic inflammation and mechanical stress.^6^ As such, treatment of chronic venous insufficiency and lower extremity edema is essential in the treatment and prevention of this condition. Treatment ranges from conservative to surgical. Patients begin with leg elevation, compression garments, and/or weight loss (if applicable) and could progress to venous ablation, vein stripping, or sclerotherapy, if conservative measures are not successful.^18^

Routine fluorescent imaging can help determine the bacterial load and identify HO lesions in the dermal and subdermal areas of the skin. We found that routine fluorescent imaging with our MolecuLight camera (MolecuLight Inc) can also help determine the bacterial load and identify HO lesions in the dermal and subdermal areas of the skin. As noted in the images from our three patients, areas with known calcifications demonstrated fluorescence on imaging. The color of fluorescence has been shown to correlate with certain bacterial species, with red indicating porphyrin-producing bacteria such as *S. aureus* and cyan indicating *P. aeruginosa*.^19^^,^^20^ This can provide a useful adjunct to wound care specialists with similar imaging capabilities; however, more study is needed.

Caution in the workup to search for other diagnoses or an inciting cause is advised. A holistic approach to healing these wounds could be necessary to help optimize closure. This would include addressing factors that could put the patient at greater risk of poor wound healing such as health literacy, access to treatment, cost of treatment, and aversion to treatment due to pain at the wound site.^21^ Aggressive management of comorbidities and early recognition of lower extremity calcifications are important. Local wound care includes proper hygiene with excisional and mechanical debridement, if needed. Fluorescent imaging can help determine further management of care. Our patients had a delay of treatment before referral to our wound center. The treatment options are limited once extensive calcifications are present. Early identification of HO is recommended. A poor prognosis of wound healing for these ulcerations can ensue, especially for patients with extensive calcifications.

## Conclusions

HO can be a complication of chronic venous insufficiency and ulcerations. Most patients have a prolonged course of a nonhealing ulceration. A missed diagnosis is common. However, routine physical examination, fluorescent imaging, radiography, CT, and/or MRI can confirm the diagnosis. The risk of osteomyelitis should be considered and, if present, treated. Biopsy of calcifications can be necessary to determine the presence of underlying osteomyelitis. Prolonged and poor wound healing is eminent without wide local excision.

## Disclosures

None.
